# Argininosuccinate synthase 1 suppresses tumor progression through activation of PERK/eIF2α/ATF4/CHOP axis in hepatocellular carcinoma

**DOI:** 10.1186/s13046-021-01912-y

**Published:** 2021-04-10

**Authors:** Sanghwa Kim, Minji Lee, Yeonhwa Song, Su-Yeon Lee, Inhee Choi, I-Seul Park, Jiho Kim, Jin-sun Kim, Kang mo Kim, Haeng Ran Seo

**Affiliations:** 1 Cancer Biology Research Laboratory, Institut Pasteur Korea, 16, Daewangpangyo-ro 712 beon-gil, Bundang-gu, Seongnam-si, Gyeonggi-do 463-400 Republic of Korea; 2 Medicinal Chemistry, Institut Pasteur Korea, 16, Daewangpangyo-ro 712 beon-gil, Bundang-gu, Seongnam-si, Gyeonggi-do 13488 South Korea; 3 Screening Discovery Platform, Institut Pasteur Korea, 16, Daewangpangyo-ro 712 beon-gil, Bundang-gu, Seongnam-si, Gyeonggi-do 13488 South Korea; 4 Department of Gastroenterology, Asan Liver Center, Asan Medical Center, University of Ulsan College of Medicine, Olympic-ro 43-gil, Songpa-gu, Seoul, 05505 South Korea

**Keywords:** Hepatocellular carcinoma (HCC), Argininosuccinate synthase 1(ASS1), Endoplasmic reticulum (ER) stress, Spheroids, C/EBP homologous protein (CHOP)

## Abstract

**Background:**

Hepatocellular carcinoma (HCC) is one of the most common malignant cancers worldwide, and liver cancer has increased in mortality due to liver cancer because it was detected at an advanced stages in patients with liver dysfunction, making HCC a lethal cancer. Accordingly, we aim to new targets for HCC drug discovery using HCC tumor spheroids.

**Methods:**

Our comparative proteomic analysis of HCC cells grown in culture as monolayers (2D) and spheroids (3D) revealed that argininosuccinate synthase 1 (ASS1) expression was higher in 3D cells than in 2D cells due to upregulated endoplasmic reticulum (ER) stress responses. We investigated the clinical value of ASS1 in Korean patients with HCC. The mechanism underlying ASS1-mediated tumor suppression was investigated in HCC spheroids. ASS1-mediated improvement of chemotherapy efficiency was observed using high content screening in an HCC xenograft mouse model.

**Results:**

Studies of tumor tissue from Korean HCC patients showed that, although ASS1 expression was low in most samples, high levels of ASS1 were associated with favorable overall survival of patients. Here, we found that bidirectional interactions between ASS1 ER stress responses in HCC-derived multicellular tumor spheroids can limit HCC progression. ASS1 overexpression effectively inhibited tumor growth and enhanced the efficacy of in vitro and in vivo anti-HCC combination chemotherapy via activation of the PERK/eIF2α/ATF4/CHOP axis, but was not dependent on the status of p53 and arginine metabolism.

**Conclusions:**

These results demonstrate the critical functional roles for the arginine metabolism–independent tumor suppressor activity of ASS1 in HCC and suggest that upregulating ASS1 in these tumors is a potential strategy in HCC cells with low ASS1 expression.

**Supplementary Information:**

The online version contains supplementary material available at 10.1186/s13046-021-01912-y.

## Background

Cancers are still the leading cause of human death, and hepatocellular carcinoma (HCC) is one of the most serious forms of cancer. The highest incidence occurs in Eastern Asia and sub-Saharan Africa. Specifically, South Korea ranks highest in the world in liver cancer incidence and mortality rate. Typical first-line therapy for HCC involves surgical resection of the tumor, whereas second-line therapy often includes sorafenib [1], a multi-tyrosine protein kinase inhibitor for treating metastatic or unresectable HCC. However, neither approach is entirely effective against HCC. Therefore, researchers have tried to identify novel target genes and drug candidates for HCC treatment, although none of these developments has yet to significantly improve patient prognoses.

Metabolic alterations in cancer cells are numerous and include aerobic glycolysis, reduced oxidative phosphorylation, and increased generation of biosynthetic intermediates needed for cell growth and proliferation. Recent data have also demonstrated that cancer cells exhibit altered amino acid metabolism [2], and glutamine, serine, glycine, and arginine have been implicated in promoting cancer cell proliferation [3–5]. In particular, arginine is associated with numerous metabolic pathways pertinent to tumorigenesis, including nitric oxide, creatine, and polyamine synthesis [6, 7].

Several types of tumors have aberrant arginine-metabolic enzymes and rely completely on extracellular arginine to support necessary biological processes, a requirement known as arginine auxotrophy. Many tumors, including HCC, malignant melanoma, malignant pleural mesothelioma, and prostate and renal cancers, are arginine auxotrophic due to their variable loss of argininosuccinate synthase 1 (ASS1), which is severely reduced or absent in numerous types of aggressive and chemoresistant cancers. However, the mechanism of ASS1 downregulation in these tumors has not been fully elucidated in HCC.

The tumor microenvironment (TME) has important physiological roles in cellular differentiation, tumorigenesis, metastasis, and therapeutic efficacy. Presently, two-dimensional (2D) cell-based models fail to predict *in vivo* efficacy, contributing to limited success in translating new drugs for clinical use. Hence, 2D culture systems alone are not beneficial because the resulting data cannot be utilized for translational research. In contrast, a complex three-dimensional (3D) cell culture system better simulates cellular context and the therapeutically relevant parameters of the in vivo TME, such as pH, oxygen level, metabolite gradients, growth factor penetration, and distribution of proliferating/necrotic cells [8, 9]. In particular, liver cells in a 3D culture system better recapitulate numerous physiological liver functions, including albumin and urea synthesis, bile secretion, and cell polarization [10, 11].

In our study, we compared the proteomes of HCC cells grown in culture as monolayers (2D) or spheroids (3D) to identify a differential global protein response under these in vitro conditions. ASS1 expression was higher in HCC cells in the 3D culture system than in the 2D system, which illustrates the importance of 3D culture in cancer biologic studies and implicates ASS1 as a new target for anti-HCC therapeutics. Moreover, we observed that low ASS1 expression in HCC tissue had a significant effect on the overall survival of patients with liver cancer. We also found that bidirectional interactions between ASS1 and ER stress responses in HCC spheroids modulated HCC cell apoptosis independent of arginine metabolism. Subsequently, we sought to identify compounds that regulate ASS1 expression to improve HCC therapy.

## Materials and methods

### Chemical agents

Endoplasmic reticulum stress inducers, including thapsigargin; TG (T9033) and tunicamycin; TM (T7765), cisplatin (C2210000) and the nitric oxide (NO) scavengers such as carboxy-PTIO potassium salt; cPTIO (C221) and Sodium diethyldithiocarbamate trihydrate; Cupral (D3506) were purchased from Sigma-Aldrich (St. Louis, MO, USA). The DNA methyltransferase inhibitor; decitabine (S1200) was purchased from Selleck Chemicals (Houston, TX, USA).

### Cell lines and cultures

The HCC cell lines ;SNU449, SNU475, SNU398, SNU898, Huh7, HepG2, Hep3B and PLC/PRF/5 were purchased from the Korean Cell Line Bank. Huh6 cells were kindly provided by Dr. Ralf Bartenschlager (University of Heidelberg, Germany). All HCC cells were maintained in RPMI (Welgene, Korea) or Dulbecco’s Modified Eagle Medium (DMEM; Welgene, Korea) containing 10% fetal bovine serum (FBS; Gibco, Grand Island, NY, USA) and 1% penicillin/streptomycin solution (p/s; Gibco, Grand Island, NY, USA). Fa2N-4, a human immortalized hepatocyte cell line, was obtained from Xenotech (Lenexa, KS, USA) and first cultured in serum-containing plating medium (K4000; Xenotech). Miha. Cells were kindly provided by Dr. Kim. K. M. from ASAN medical center. To produce tumor spheroids, HCC cells seeded at a density of 6 × 10^3^ cells/well in 96-well round-bottom ULA microplates (Corning Life Sciences, Corning, NY, USA). All cells were maintained in a 5% CO_2_ humidified incubator at 37 °C.

### Ethics approval and consent to participate

This study was conducted in accordance with the Declaration of Helsinki, and samples were provided from patients of ASAN medical center. Approximately 60 patients with a diagnosis of liver cancer who were treated and followed up in the clinic participated. The study was approved by the Human Research Ethic Committee of ASAN medical center (Permit Number: 2007–0332). The Institutional Review Board of ASAN medical center complies with the related laws including ICH, KGCP, and the Bioethics and Safety Act of Korea. Written informed consent for the use of tissues for research purposes was obtained from patients at the time of tumor specimen procurement.

### Primary culture of HCC-derived cells

We acquired a portion of human HCC tumors immediately after surgical resection. The tumors were immersed in Hanks’ balanced salt solution (Gibco-ThermoFisher, Waltham, MA, USA). Patient-derived primary HCC cell lines (17S-35372B, 17S-64007, 17S-68354, 17S-21612B, 17S-121312B, 27273, 101509, 103201,  and 116261) were generated from liver cancer tissues of nine Korean patients.

### Proteomic analysis

Monolayer (2D) and tumor spheroid (3D) lysates from patient-derived primary cells were separated and analyzed using nanoACQUITY UPLC system (Waters, Milford, MA, USA) directly coupled to a Finnigan LCQ DECA ion trap mass spectrometer (Thermo Fisher). The samples were analyzed using a MS/MS spectra system (Thermo Quest, San Jose, CA, USA) and processed using SEQUEST software purchased from Thermo Fisher.

### Microarray and bioinformatics analysis

From the generated microarray data, we selected genes identified by FUNRICH (functional enrichment analysis tool; http://www.funrich.org) and REACTOME database pathway whose expression levels changed ≥2-fold (absolute) when enriched by ASS1-overexpressing HCC spheroids. We then determined if these genes were enriched or depleted in molecular functions, physiological processes, and biological pathways.

### Western blot assay

Cell pellets were lysed by RIPA buffer (CureBio, Seoul, Korea) for 30 min at 4 °C, and lysates were collected by centrifugation at 20,000 g for 30 min. The protein concentration of each group’s cell lysate was measured using the Pierce™ BCA protein Assay Kit (ThermoFisher, Cambridge, MA, USA). Equal amounts of proteins were separated on an SDS-PAGE gel using the electrophoretic technique. The gel was transferred to a nitrocellulose membrane using a BioRad transfer system (Hercules, CA, USA) and blocked with 5% skim milk. Primary antibodies for hybridization included those for ATF6 (65880S), IRE1α (3294S), ATF3 (33593S), GRP78 (3117S), cleaved caspase 3 (7237S), PARP (5625S), Snail (3879S) from Cell Signaling Technology (Danvers, MA, USA). The antibodies of ASS1 (ab124665), CHOP (ab11419), XBP1 (ab37152), α-SMA (ab32575), N-cadherin (ab76057), E-cadherin (ab15148) are purchased from Abcam (Cambridge, UK), and β-actin (A5441) from Sigma-Aldrich (St Louis, MO, USA). Secondary antibodies conjugated with horseradish peroxidase were then applied to the blots. Resulting protein bands were detected using the LuminoGraph II system (ATTO, Tokyo, Japan).

### Immunoprecipitation (IP) analysis

The cell lysates were harvested and lysed with lysis buffer containing protease inhibitors. The cell extracts (2 mg/500 μl) were conducted immunoprecipitation using Pierce™ Co-immunoprecipitation Kit (Thermo Fisher, Cambridge, MA, USA) following to manufacturer’s instructions. The samples were incubated with anti-Flag (Thermo Fisher) at 4 °C overnight, and conducted by western blot assay.

### Immunofluorescence (IF)

For immunofluorescence, the samples were fixed in 4% paraformaldehyde (PFA) and washed in DPBS with Tween 20 (DPBS-T). After than the samples were blocked in DPBS-T plus 10% NGS (normal goat serum) for 1 hr at room temperature(RT). Samples were then incubated with primary antibodies and secondary fluorophore-conjugated antibodies. Cell nuclei were stained using Hoechst 33342 (1:1000, MOP-H3570; ThermoFisher, Cambridge, MA, USA). After washing, the samples were mounted, and fluorescence images were detected using an automated high-content imaging system (OPERETTA, PerkinElmer, Waltham, MA, USA) and confocal laser scanning microscope (CLSM II; LSM710A, Carl Zeiss, Germany). The results were analyzed by an in-house software tool and HARMONY 3.5.1. (PerkinElmer, Waltham, MA, USA).

### Transfection with siRNAs

Huh7 cells were seeded at 5 × 10^5^ cells in 10mm dish. siRNA targeting ASS1 were purchased from Dharmacon (Lafayette, CO, USA; L-004819-00-0005, SMARTpool:ON-TARGETplus ASS1 siRNA, USA), Bioneer (Daejeon, Korea; siRNA ID:445–1, 445–2 and 445–3) and Santa Cruz Biotechnology (sc-45,810, Santa Cruz Biotechnology Inc., Dallus, TX, USA). siRNA targeting CHOP were purchased from Dharmacon (Lafayette, CO, USA; L-010257-00-0005, SMARTpool:ON-TARGETplus CHOP siRNA, Lafayette, CO, USA). siRNAs were transfected into HCC cells using Lipofectamine RNAiMAX (Invitrogen, Carlsbad, CA, USA) reagent with Opti-MEM, and the cells were incubated in 37 °C for 48 hr.

### Cell survival and apoptosis analysis assay

To measure the cell viability, ATP level was analyzed using the Cell Titer-Glo luminescent cell viability assay kit (Promega, Madison WI, USA). To analyze apoptosis capacity, Caspase3/7 activity and Annexin V activity were measured using the Caspase-Glo 3/7 assay kit (Promega, Madison, WI, USA) and Real-time-Glo™ Annexin V Apoptosis Assay kit (Promega, Madison, WI, USA) following manufacturer’s instructions, repectively.

### Wound healing and colony formation assays

Huh7 cells were transfected with pCMV3 or ASS1-Flag (overexpression) were seeded (1 × 10^6^ cells/well) in RPMI with 10% FBS in 6-well plates. After 24 hr, cells were grown to full confluence in plates and scratched using a pipette tip. Cells were washed with PBS to remove cellular debris and allowed to migrate for 24 h. Cell migration images were captured using light microscopy, and five fields in the wounded areas were noted. For colony formation assays, 1 × 10^3^ Huh7, SNU475, and SNU449 cells were transfected with pCMV3 or ASS1-Flag and seeded (1X10^3^ cells/well) in 6-well plates. Starting the day after seeding, the medium was replaced every 3 days. The cells were cultured for 14 days, and then the plates were washed with Dulbecco’s PBS (DPBS) and stained with crystal violet (Sigma-Aldrich, St Louis, MO, USA). The number of colonies were counted and analyzed.

### NO production assay

To measure the NO production levels, HCC cells that transfected with ASS1-flag or siASS1 were seeded at 96-well flat-bottom enzymatic assay plate. NO level was measured using Griess Reagent System kit (Promega, Madison, WI, USA) following manufacturer’s instructions. The Nitrite oxidant was measured absorbance within 30 min in a plate reader with a filter between 520 nm and 550 nm.

### Dose-response curve experiments

Huh7, SNU475 and Hep3B cells transfected with pCMW3, ASS1-Flag, or siRNA agaist ASS1 were seeded in 384-well plates at a density of 2 × 10^3^ cells/well. For single treatment, the concentration of compounds including cisplatin, thapsigargin, and NO scavengers (cPTIO and cupral) was diluted from 10 μM to 39.06 nM in 0.5% DMSO (v/v). After 48 hr, the cells were fixed in 4% paraformaldehyde (PFA) for 10 min and washed twice with Dulbecco’s PBS (DPBS). Cell nuclei were stained using Hoechst 33342. To detect an enough cells using an automated high-content imaging system (OPERETTA, PerkinElmer, Waltham, MA, USA), five field images were captured and collected from each well. The images were analyzed by an in-house software tool and HARMONY 3.5.1. (PerkinElmer, Waltham, MA, USA).

### Luciferase assay

To construct an ASS1-Gluc stable cell line, HEK293 cells were transfected with the ASS1 promoter and firefly luciferase gene sequence in the pEZX-PG02.1 lentiviral vector. Transduced cells were selected using puromycin. After constructing the stable cell line, cells were seeded onto 96-well plates. The luciferase reporter assay was conducted with the Nano-Glo® Luciferase Assay System (Promega, Madison, WI, USA) according to the manufacturer’s instructions. The luminescence from firefly and Renilla luciferase were measured by a luminometer (Berthold, Bad Wildbad, and Germany).

### Xenograft mouse model

Huh7 cells (5 × 10^6^ cells/mouse) were transplanted following resuspension with Matrigel (reduced matrix growth factor reduced; BD Biosciences) and orthotopically injected into 5-week-old male BALB/c nude mice (Central Lab. Animal, Inc., Seoul, Korea). The mice kept in a laboratory animal facility with a constant temperature of 20 °C ± 2 °C and relative humidity of 50% ± 10% under regular light-dark cycle. At 2 weeks post-injection, the mice were randomly divided into 6 groups (5 mice per group): 1) control (saline), 2) cisplatin 5 mpk (mg/kg body weight), 3) decitabine 1 mpk, 4) decitabine 2 mpk, and combination groups including 5) cisplatin 5 mpk + decitabine 1 mpk and 6) cisplatin 5 mpk + decitabine 2 mpk. Each compound or combination was then administered for 1 week by I.P. injection, after which the mice were sacrificed. Throughout the experimental period, body weight and tumor size were measured twice a week using calipers and calculated tumor volume (mm^3^) by the standard formula. The experiment was approved by the Institutional Animal Care and Use Committee (IACUC) of ASAN medical center.

### Statistical analysis

The statistical significance between groups was determined using the Student’s *t*-test with a Newman-Keuls post-hoc test using Prism 8 (GraphPad, San Diego, CA, USA). *P* < 0.05 was considered statistically significant.

## Results

### The expression of ASS1 is upregulated in HCC spheroids

**Fig. 1:**
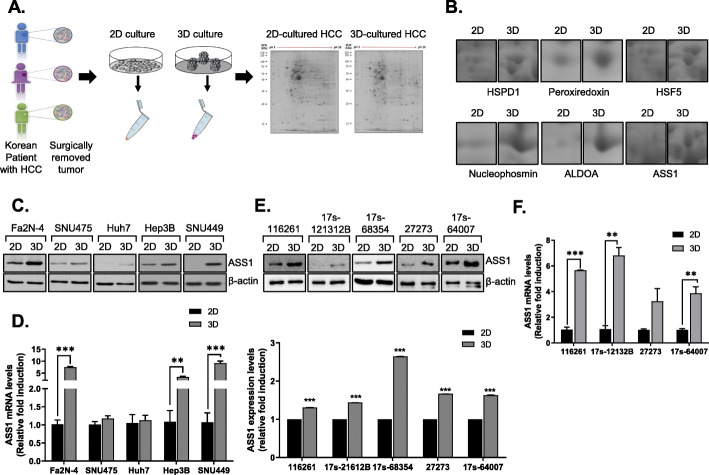
Expression of ASS1 is upregulated in HCC spheroids **a** Schematic of the proteomics analysis of Korean patient-derived HCC cells cultured in monolayers (2D) or spheroids (3D). **b** Expression of identified proteins including HSPD1, peroxiredoxin, HSF5, Nucleophosmin, ALDOA, and ASS1 in 2D or 3D-cultured HCC. Expression of ASS1 in 2D or 3D-culured system from Fa2N-4 and HCC cell lines by **c** Western blot assay and **d** qRT-PCR. Expression of ASS1 in patient-derived HCC cell lines **e** Western blot and **f** qRT-PCR. ^**^P < 0.01 and ^***^P < 0.001 compared to control groups

In order to investigate the role of cell adhesion, mediated by cell-cell and cell-ECM interactions in the tumor microenvironment, in the molecular mechanism underlying ASS1 function, we compared the proteomes of HCC cells cultured as monolayers (2D) or spheroids (3D) to identify a global protein response in the different in vitro conditions (Fig. 1a). We focused on polypeptides upregulated by at least 4-fold in HCC spheroids relative to monolayers (*P* ≤ 0.05). Specifically, six proteins (nucleophosmin, peroxiredoxin, HSF5, aldolase A, HSPD1, and ASS1) were expressed at a higher level in HCC cell spheroids than in monolayers (Fig. 1b). Thus, we investigated the expression patterns of these proteins in HCC monolayers and spheroids.

Western blot analysis revealed that aldolase A, HSPD1, and ASS1, but not nucleophosmin and peroxiredoxin, were significantly increased in HCC spheroids compared to monolayers. HSF5 was not detected by western blotting in HCC monolayers or tumor spheroids (Additional file 5: Fig. S1 and Fig. 1c). Because metabolic alterations have been highlighted recently as targets for HCC therapy, we focused on the metabolism-related protein ASS1 among HCC spheroid-specific proteins.

To confirm whether ASS1 expression is cell adhesion status - dependent, we measured ASS1 protein expression in lysates of monolayers and spheroids of HCC cell lines including Huh7, Hep3B, SNU475 and SNU449 and of Fa2N-4 normal hepatocytes. In the monolayer culture system, the HCC cell lines scarcely expressed ASS1, whereas Fa2N-4 cells showed higher ASS1 expression. Interestingly, ASS1, which was minimally expressed in HCC monolayers, was highly expressed in HCC spheroids (Fig. 1c) and *ASS1* mRNA expression was also altered in a culture-type–dependent manner (Fig. 1d).

To avoid using the less physiologically relevant genetically defined human cell lines, we attempted to confirm increased ASS1 expression in spheroids cultured using primary HCC cells isolated from liver resection specimens of five patients. We measured ASS1 mRNA and protein in lysates of monolayers and spheroids cultured from patient-derived primary HCC cells. Similar to the results with HCC cell lines, ASS1 mRNA and protein were expressed at higher levels in patient-derived HCC spheroids than in monolayers (Fig. 1e, f). These results suggested that ASS1 expression is upregulated in the 3D HCC tumor microenvironment.

### HCC patients expressing higher levels of ASS1 have a more favorable prognosis

**Fig. 2:**
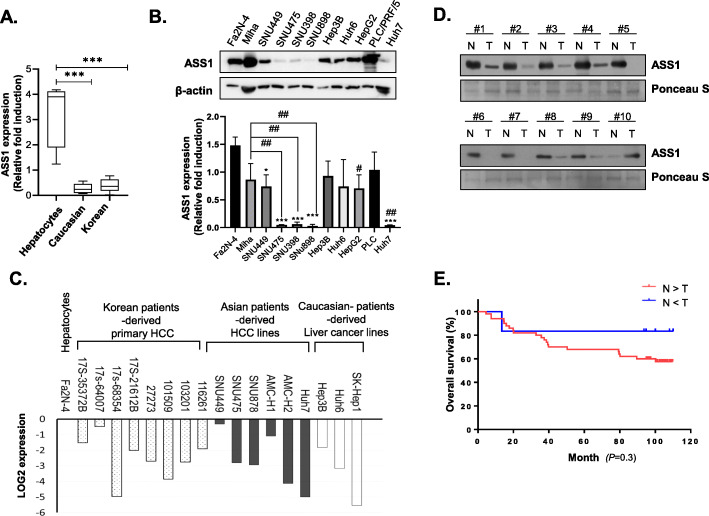
Patients with increased ASS1 expression in HCC have a more favorable prognosis **a** ASS1 mRNA expression levels and **b** Expression of ASS1 in hepatocytes; Fa2N-4, miha., Caucasian-derived HCC cells; Hep3B, Huh6, HepG2, and PLC/PRF/5, and Korean-derived HCC cells; SNU449, SNU475, SNU398, and SNU898. **c** ASS1 mRNA expression levels in tumor spheroids from eight Korean patient-derived HCC cell lines, six Asian patient-derived HCC cell lines (SNU449, SNU475, SNU878, Huh7, AMC-H1 and AMC-H2), three Caucasian patient-derived HCC cell lines (Hep3B, Huh6 and SKhep-1), and normal hepatocyte (Fa2N-4). **d** ASS1 expression in adjacent peritumoral tissues (N) and tumor tissues (T) from HCC patient samples by western blot analysis. **e** 10-year overall survival rates of patients with high *vs.* low expression of ASS1. (N = peritumoral tissues, T = tumor tissues) ^*^P < 0.05 and ^***^P < 0.001 compared to control group

Epigenetic silencing via methylation of the *ASS1* promoter was previously demonstrated in certain cancer types, so we assessed *ASS1* mRNA levels in immortalized normal hepatocyte (Fa2N-4 and Miha cell) spheroids and in HCC spheroids derived from Asian (SUN449, SUN475, SNU878, and SNU398 cells) and Caucasian (Hep3B, Huh6, HepG2, and PLC/PRF/5 cells) patients. Reverse transcription (RT)-quantitative (q) PCR analyses showed that all HCC spheroids express lower levels of ASS1 than spheroids of normal hepatocytes (Fig. 2a). Next, we also examined ASS1 protein expression in HCC spheroids derived from Asian and Caucasian HCC patients. Although previous studies had been confirmed ASS1 expression levels in various HCC cell lines [12], we additionally conducted some of Korean patient-derived HCC cell lines and normal hepatocytes and classified as Korean patients –derived HCC and Caucasian patients-derived HCC cell lines. As the results, Korean patient-derived HCC cells showed dramatically lower ASS1 expression than spheroids of normal hepatocytes. In contrast, some spheroids of Caucasian patient-derived liver cancer lines, such as Hep3B and PLC/PRF/5, exhibited ASS1 expression similar to that of normal hepatocytes. The ASS1 expression appears to be lower in HCC cells derived from Asian patients than from Caucasian patients (Fig. 2b).

We investigated *ASS1* mRNA expression in tumor spheroids from primary HCC cells derived from eight Korean liver cancer patients. The cells were passaged only 3–6 times. We also measured *ASS1* mRNA expression in tumor spheroids from six Asian patient-derived HCC lines (SUN449, SUN475, SNU878, AMC-H1, AMC-H2, and Huh7), three Caucasian patient-derived liver cancer lines (Hep3B, Huh6, and adenocarcinoma line; SK-Hep1), and normal hepatocyte (Fa2N-4). All tumor spheroids of the eight Korean patients displayed significantly lower *ASS1* mRNA expression than in spheroids of normal hepatocytes. Expression of *ASS1* mRNA was also lower in HCC spheroids of Caucasian and Asian patients than in spheroids of normal hepatocyte (Fig. 2c).

Based on these results, we next investigated the clinical significance of ASS1 expression in Korean patients with liver cancer because its incidence and mortality rate is the highest in the world, even though ASS1 is not prognostic in liver cancer according to The Cancer Genome Atlas (TCGA) program; ‘https: //www.proteinatlas.org/ENSG00000130707-ASS1’

ASS1 expression was quantified by western blotting in tissue from 58 Korean patients with nodular liver cancer and hepatitis B infection. Representative ASS1 expression patterns are shown in Fig. 2d and Additional file 6: Fig. S2.

Because ASS1 expression varied among patients, we performed further statistical analysis. The 58 liver cancer patients were divided into three groups based on the results of western analysis: 1) higher ASS1 expression in tumor vs. peritumoral tissue (*n* = 7); 2) lower ASS1 expression in tumor vs. peritumoral tissue (*n* = 51); and 3) no significant difference in ASS1 expression between tumor and nontumor tissue (*n* = 0). The results of this analysis indicated that ASS1 expression was lower in tumor tissue than peritumoral tissue from Korean HCC patients. We wondered whether a correlation existed between ASS1 expression in tumor tissue and survival rate after resection in patients with liver cancer. The two groups with differential ASS1 expression exhibited significant differences in 10-year survival rates after resection that were significantly higher when ASS1 expression was higher in tumor vs. peritumoral tissues (Fig. 2e).

These results suggest that HCC patients with increased ASS1 expression in tumor tissue have a more favorable prognosis than patients with lower ASS1 expression, which prompted us to focus on the potential tumor suppressor roles of ASS1 during HCC progression.

### ASS overexpression inhibits HCC tumor growth and improves chemotherapy efficacy

**Fig. 3:**
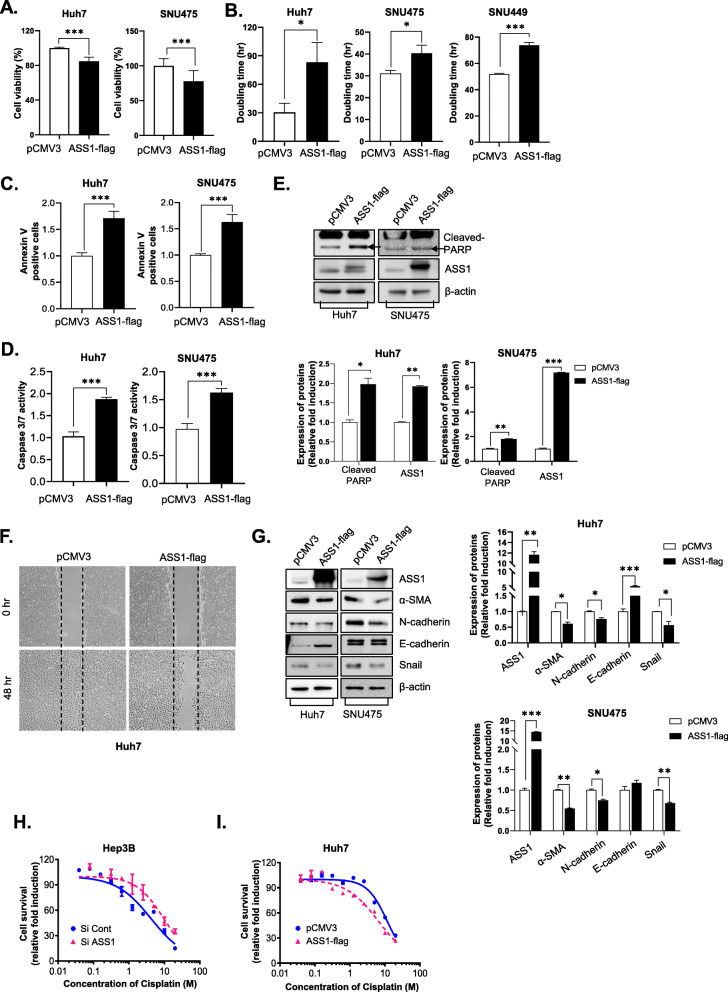
Overexpression of ASS1 inhibits HCC tumor growth and improves chemotherapy efficiency **a** Cell viability analysis of Huh7 and SNU475 and **b** Doubling time analysis of Huh7, SNU475 and SNU449 cells transfected with pCMV3 or ASS1-flag. The measurement of **c** Annexin V staining apoptosis analysis, **d** Caspase3/7 activity and **e** Expression of cleaved PARP (upper panel) and the quantitative analysis graph (bottom panel) in Huh7 and SNU475 cells transfected with pCMV3 or ASS1-Flag. **f** Wound healing assays of Huh7 cells transfected with pCMV3 or ASS1-Flag. **g** Expression of EMT-related proteins, including a-SMA, N-cadherin, E-cadherin, and Snail (left panel) and the quantitative analysis graph (right panel) in Huh7 and SNU475 cells transfected with pCMV3 or ASS1-Flag. Cell survival curves of **h** siRNA against ASS1-transfected in Hep3B cells and **i** ASS1 transfected in Huh7 cells treated with cisplatin at the indicated concentrations. ^*^P < 0.05, ^**^P < 0.01 and ^***^P < 0.001 compared to control group

To investigate the effects of altered ASS1 expression on HCC cell growth and migration, we established ASS1-overexpressing HCC cell lines. First, we examined whether ASS1 controlled cell growth in HCC. Cell survival was diminished by ASS1 overexpression in Huh7 and SNU475 cells (Fig. 3a), whereas the cell doubling times increased in Huh7, SNU475 and SNU449 cells (Fig. 3b).

To determine whether the ASS1-induced inhibition of cell growth was associated with an increase in apoptosis, we evaluated apoptosis-related parameters using Annexin apoptosis assay kit. Following ASS1 overexpression, the number of Annexin V-positive Huh7 and SNU475 cells was increased (Fig. 3c). Additionally, we measured caspase-3/7 activity and expression of cleaved poly (ADP-ribose) polymerase (PARP) in ASS1-overexpressing HCCs. ASS1 overexpression not only promoted caspase-3/7 activity (Fig. 3d), but also increased levels of cleaved PARP in Huh7 and SNU475 cells (Fig. 3e). These results showed that ASS1 overexpression can inhibit HCC growth by delaying cell growth and inducing apoptosis.

Wound-healing assays revealed that the migratory capacity of HCC cells was attenuated by ASS1 expression (Fig. 3f). Because cells in epithelial-to-mesenchymal transition (EMT) acquire increased migratory capacity, we next measured the expression of EMT-related proteins (E-cadherin, N-cadherin, α-SMA, and Snail) in ASS1-overexpressing HCC cells. As the result, the expression of mesenchymal-like markers such as N-cadherin, Snail, and α-SMA were downregulated by ASS1-overexpression. While the expression of E-cadherin that is epithelial marker was elevated in ASS1-overexpressed HCC cells. Therefore, EMT could be inhibit by ASS1 overexpressed Huh7 and SNU475 cells, that suggested ASS1 plays a pivotal role EMT during HCC progression (Fig. 3g).

Previous reports showed that cisplatin sensitivity is restricted during cancer in an ASS1- expression–dependent manner [13, 14]. Herein, we observed responses to cisplatin with respect to ASS1 expression levels in Hep3B and PLC/PRF/5 cells, which normally express higher than average levels of ASS1 among HCC cell lines, and Huh7 and SNU475 cells, which minimally express ASS1. When we compared the sensitivities of normal and ASS1-deficient Hep3B cells to cisplatin treatment coupled with siRNA-mediated ASS1 knockdown, depletion of ASS1 shifted the cisplatin IC_50_ from 4.231 μM to 9.372 μM (Fig. 3h). Moreover, ASS1-deficient PLC/PRF/5 cells showed slight resistance to cisplatin relative to control-siRNA-transfected cells (Additional file 2: Table S2), whereas ASS1-overexpressing Huh7 and SNU475 cells displayed greater sensitivity to cisplatin than wild-type cells (Fig. 3i, Additional file 3: Table S3). Furthermore, sensitivity to sorafenib, which is the only systemic chemotherapeutic agent available for HCC, was slightly affected by altered ASS1 expression [Additional file 2: Tables S2 and Additional file 3: Table S3].

### ASS1 is upregulated through ER stress responses in HCC spheroids

**Fig. 4:**
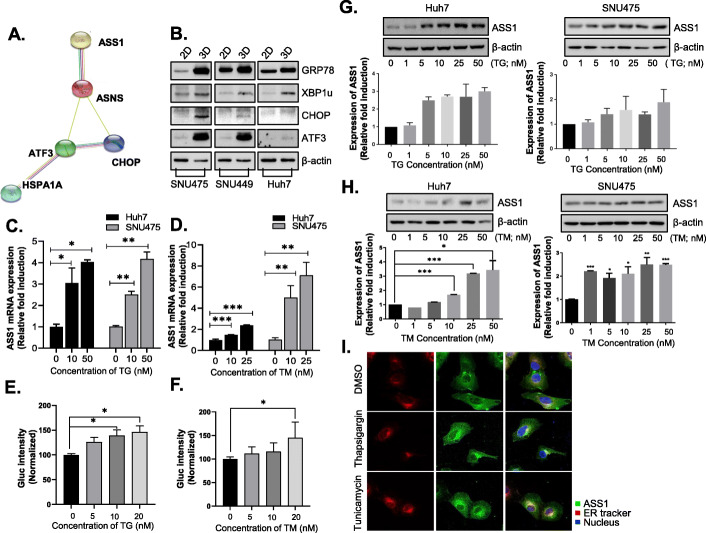
ASS1 is upregulated through ER stress response in HCC spheroids (**a**) Microarray analysis of ASS1-overexpressing HCC spheroids implicated four genes for further study (*ASNS*, *ATF3*, CHOP, and *HSPA1A*). (**b)** Expression of ER stress response related proteins; GRP78, XBP1us, CHOP and ATF3 in monolayers (2D) or spheroids (3D) of HCC cell lines including SNU475, SNU449 and Huh7 cells. (**c-d**) ASS1 mRNA levels analysis in Huh7 cells and SNU475 cells with treatment of ER stress inducers; TG and TM by qRT-PCR. (**e-f**) ASS1 promoter activity by luciferase assay in Huh7 cells with treatment of TG and TM. ASS1 expression levels in Huh7 and SNU475 cells with treatment of (**g**) TG and (**h**) TM. (**i**) Immunofluorescence (IF) images showing ASS1 (green), ER (red), and nuclei (blue) in Huh7 cells with TG and TM treatment. ^*^P < 0.05, ^**^P < 0.01 and ^***^P < 0.001 compared to control group. At least triplicate analysis in each set of experiment. TG; thapsigargin, TM; tunicamycin

To identify genes associated with ASS1 upregulation, we performed microarray analysis using ASS1-overexpressing HCC spheroids. Four genes, *ASNS*, *ATF3*, *CHOP*, and *HSPA1A*, were selected because their expression levels changed ≥2-fold with ASS1 overexpression in HCC cells. Based on the results of pathway analysis *ATF4*, *ATF6*, *HSP90B1*, *HSPA5*, *CALR*, and *XBP1* were also examined for their roles in ASS1 signaling (Fig. 4a, Additional file 1: Table S1).

Because most of the genes selected from the microarray analysis were closely related to potential ER stress responses, and the mechanism of ASS1 overexpression in spheroids was unknown, we explored the possible correlation between ER stress response and ASS1 expression in HCC monolayers or HCC spheroids. As expected, HCC spheroids of SNU475, SNU449, and Huh7 cells exhibited higher expression levels of ER stress-response–related proteins (CHOP, XBP1u, GRP78, and ATF3) and ASS1 than monolayer cells (Fig. 4b).

To determine whether ER stress contributes to ASS1 synthesis in HCC microenvironments, we analyzed the effects of thapsigargin (TG) and tunicamycin (TM), which induce ER stress and the unfolded protein response (UPR), on regulation of ASS1 expression in Huh7 and SNU475 cells. *ASS1* mRNA expression significantly increased in a dose-dependent manner with TG (Fig. 4c) and TM (Fig. 4d) treatment in Huh7 and SNU475 cells by enhancing ASS1 promoter activity (Fig. 4e, f). Accordingly, treatment with TG and TM resulted in increasing ASS1 protein expression in Huh7 and SNU475 cells (Fig. 4g, h). Furthermore, immunofluoresence assay results revealed that ASS1 is translocated from the cytoplasm to the ER during TG- or TM-induced ER stress response and UPR in HCC cells (Fig. 4i). These results suggested that ASS1 is upregulated via ER stress response in the HCC cell lines including Huh7 and SNU475 cells.

### ASS1 induced cell death through upregulation of ER stress related proteins in HCC

**Fig. 5:**
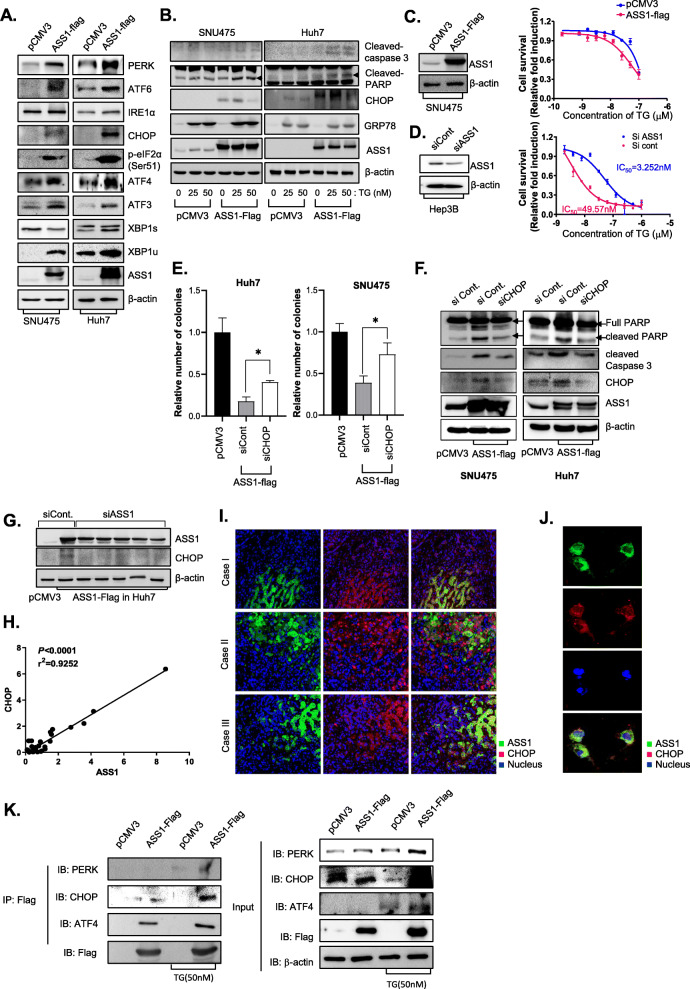
ASS1 controls cell fate through upregulation of CHOP in HCC **a** Expression of ER stress-related proteins; PERK, ATF6, IRE1a, CHOP, ATF3 XBP1s and XBP1u in pCMV3 or ASS1-flag-transfected Huh7 and SNU475 cells. **b** Expression of apoptosis-related proteins; PARP and caspase 3 active forms and ER stress related proteins; CHOP and GRP78 in Huh7 and SNU475 cells after treatment with TG. Cell viability analysis of TG treatment in **c** pCMV3 or ASS1-flag transfected SNU475 cells and **d** siRNA against ASS1 transfected Hep3B cells. **e** The colony formation assay and **f** The expression of apoptosis related proteins in Huh7 and SNU475 cells transfected with either non-specific control siRNA (siCont.) or CHOP siRNA (siCHOP) in ASS1 overexpressed Huh7 and SNU475 cells. **g** Expression of ASS1 and CHOP in ASS1 overexpressed Huh7 cell transfected with non-specific siRNA (siCont.) or ASS1 siRNA (siASS1) by western blot assay. **h** The correlation expression levels between ASS1 and CHOP in tumor tissues from HCC patients. Immunofluorescence (IF) staining of ASS1 and CHOP in **i** Tumor tissues from patients with liver cancer and **j** Huh7 cells. **k** Co-immunoprecipitation (IP) assay of Huh7 cells transfected with ASS1-Flag. The samples were analyzed by immunoblotting with an anti-PERK, ATF4, and CHOP. *P < 0.05 compared to siControl group

We next focused on the functional roles of ASS1 with respect to ER stress response in HCC. Among three ER stress sensor proteins including inositol-requiring enzyme-1 (IRE1), PKR-like ER kinase (PERK) and qand activating transcription factor-6 (ATF6), ASS1 overexpression in Huh7 and SNU475 cells led to notable upregulation of PERK and ATF6 expression (Fig. 5a). The PERK/eIF2α/ATF4/CHOP pathway has pivotal roles in the induction of apoptotic cell death during ER stress responses [15]. ATF6 is cleaved by S1P and S2P proteases in the Golgi apparatus under ER stress conditions, and then cleavage of ATF6 (p50 ATF6f) leads to upregulation of chaperones, XBP1, and the pro-apoptotic factor CHOP via translocation to the nucleus [16]. Cancer cells exploit the IRE1α-XBP1s arm of the ER stress response to efficiently adjust their protein-folding capacity and ensure survival under hostile tumor microenvironmental conditions [17]. Interestingly, ASS1 overexpression increased expression of proteins in PERK/eIF2α/ATF4/CHOP signaling nodes and expression of XBP1u, which is induced by ATF6, whereas expression of proteins in the IRE1α-XBP1s pathway was not altered in HCCs by ASS1 overexpression.

CHOP (DDIT3) in the PERK/eIF2α/ATF4/CHOP pathway, also known as C/EBP homologous protein and DNA damage inducible transcript 3, plays a central role in ER stress-mediated apoptosis [18–20].

Because its expression was substantially increased in ASS1-overexpressing HCC cells, we explored whether ASS1 overexpression could increase ER stress-mediated apoptosis. Following TG treatment in SNU475 and Huh7 cells for 48 hr, markers of apoptosis such as cleaved PARP and cleaved caspase-3, EMT related-proteins, as well as CHOP, were significantly upregulated in ASS1-overexpressing *vs.* wild-type SNU475 and Huh7 cells (Fig. 5b, Additional file 7: Fig. S3). ASS1-overexpressing SNU475 cells displayed greater sensitivity to TG than wild-type SNU475 cells (Fig. 5c). ASS1 was also silenced in Hep3B cells to confirm whether ASS1 knockdown would inhibit ER stress-mediated apoptosis in HCC cells that innately express higher than average levels of ASS1. Compared with control- siRNA-transfected cells, ASS1 knockdown led to significantly reduced survival of cells treated with TG (Fig. 5d). These results demonstrated that ASS1 overexpression could facilitate and magnify ER stress-mediated apoptosis in HCC.

Because ASS1 overexpression concurrently elevated CHOP levels and facilitated ER stress-mediated apoptosis, we next examined whether CHOP is the key mediator of the ASS1-mediated apoptosis in HCCs.

Rescue experiments downregulating CHOP in ASS1-overexpressing HCC cells were performed using a colony-forming assay. Inhibition of CHOP restored diminished clonogenic survival by ASS1 overexpression in Huh7 and SNU475 cells (Fig. 5e). Moreover, increased levels of cleaved PARP and cleaved caspase-3 by ASS1 overexpression were also inhibited by depletion of CHOP in HCC cells (Fig. 5f). These results demonstrate that CHOP is the key mediator of ASS1-mediated apoptosis in HCCs.

We next examined whether ASS1 directly modulates CHOP expression using siRNAs targeting ASS1 in ASS1-ovexpressing Huh7 cells. Transfection with siRNA targeting ASS1 effectively attenuated expression of CHOP (Fig. 5g). To explore the relationship between ASS1 and CHOP *in vivo*, we investigated expression patterns of ASS1 and CHOP in patient HCC tissues. ASS1 protein expression was proportionally correlated with CHOP expression (Fig. 5h). The patterns of ASS1 and CHOP expression revealed by immunofluoresence staining of HCC tissues showed that the expression levels of those two proteins were not only correlated with one another but that the proteins were also co-localized (Fig. 5i). Taking a closer look, co-localization of ASS1 and CHOP was also observed in Huh7 cells (Fig. 5j). To evaluate the direct interaction between ASS1 and PERK pathway related genes including PERK, ATF4 and CHOP in response induction of ER stress, we conducted immunoprecipitation (IP) assay with anti-flag in ASS1-flag overexpressed cells. As the result, ATF4 and CHOP were dominantly interacted with ASS1, especially in response ER stress induce condition (Fig. 5k).

### ASS1 acquires tumor suppressor activity independent of arginine metabolism and the p53 pathway

**Fig. 6:**
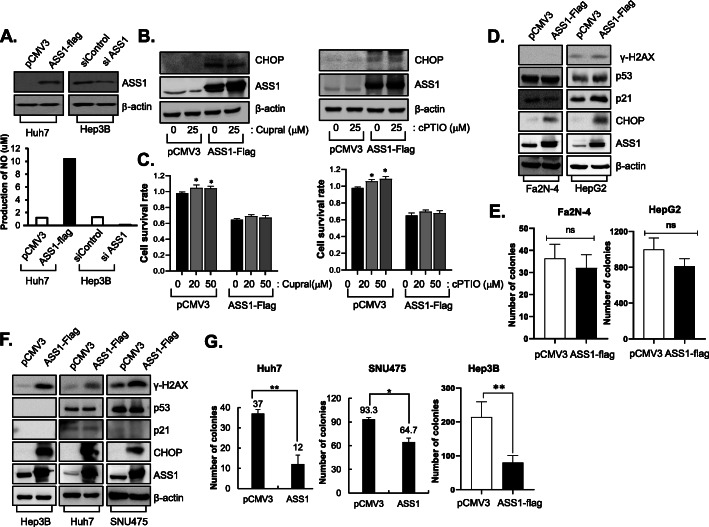
ASS1 acquires tumor suppressor activity independently of arginine metabolism and the p53 pathway **a** The NO production in ASS1-upregulation or downregulation in HCC cells. **b** ASS1 and CHOP expression levels and **c** cell survival analysis in pCMV3 or ASS1-treansfected Huh7 cells with the NO scavenger treatment; cupral (left panel) and cPTIO (right panel). **d** Phosphorylation of H2AX (γ-H2AX) and p53 pathway related proteins expression level in pCMV3- or ASS-flag transfected p53 wild type cells (Fa2N-4 and HepG2 cells) and **e** Colony formation assay in pCMV3- or ASS1-flag transfected p53-wild type cells. **f** Expression of γ-H2AX p53 related proteins in p53-null cell (Hep3B) and p53-mutant cells (Huh7 and SNU475 cells). **g** Colony formation assay in pCMV3- or ASS1-flag transfected p53-null and p53-mutant cells. ^*^P < 0.05, ^**^P < 0.01 compared to control group. At least triplicate analysis in each set of experiment. ns; not significant, NO; nictic oxide

Through arginine synthesis, ASS1 plays critical key roles in the production of nitric oxide (NO), which paradoxically exhibits both cancer-promoting and -restricting effects depending on the cellular environment. Thus, we also explored whether the effects of ASS1 on ER stress-mediated apoptosis is dependent on NO production in HCC and found that NO was increased in ASS1-overexpressing cells and was reduced in ASS1-depleted cells (Fig. 6a). We detected altered expression of CHOP in ASS1-overexpressing cells following treatment with the NO scavengers including cPTIO and cupral. Increased levels of CHOP previously detected in ASS1-overexpressing HCC cells were not altered by NO scavenging (Fig. 6b). Moreover, treatment with cPTIO and cupral did not affect inhibition of cell survival caused by ASS1 overexpression (Fig. 6c). These results indicate that ER stress-mediated apoptosis in ASS1-overexpressing cells is not related to NO production derived from arginine metabolism.

As *p53* is a well-characterized tumor suppressor gene, we determined whether ASS1-associated ER stress could regulate the p53 pathway in HCCs. ASS1 overexpression did not alter *p53* or *p21* expression in p53 wild-type HCC cells (Fa2N-4 and HepG2), as well as CHOP and phospho-histone H2AX (γ-H2AX) expression also did not change by ASS1 expression in p53 wild-type HCC cells (Fig. 6d). In addition, cell survival by ASS1 expression was not distinctly observed in p53 wild-type cells (Fig. 6e). However, expression of CHOP and γ-H2AX were dramatically increased by ASS1 overexpression exclusively in p53-mutant HCC cells (Huh7, SNU475 and Hep3B) (Fig. 6f). Moreover, clonogenic survival by ASS1 expression were significantly diminished in p53-mutant HCC cells (Fig. 6g). These results demonstrate that the ASS1-dependent DNA damage and -CHOP associated apoptosis are more facilitated in p53-mutant HCC than in p53 wild-type HCC cells.

### Decitabine improves the efficacy of anti-HCC chemotherapeutic drugs by increasing ASS1 expression

Because ASS1 plays a key role in ER stress-mediated apoptosis, we aimed to identify modulators of ASS1 expression to improve HCC therapy. Hence, we performed screening to identify compounds that specifically alter the activity of the *ASS1* promoter.

**Fig. 7:**
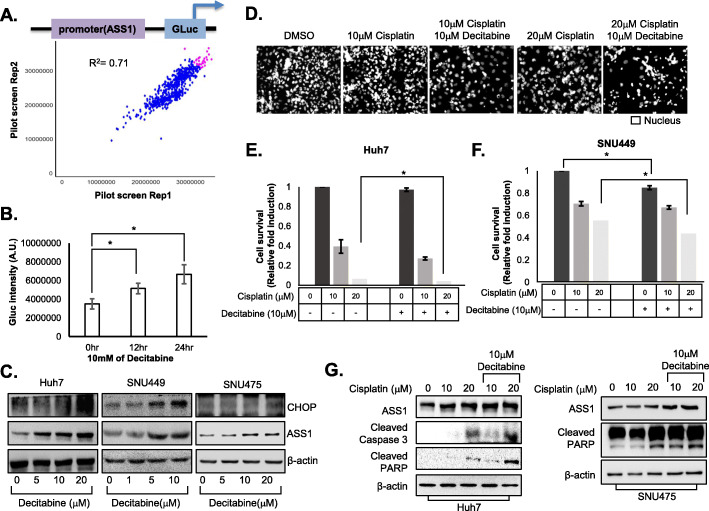
Decitabine improves efficacy of anti-HCC chemotherapeutic drugs by increasing ASS1 expression in HCC cells **a** The results of ASS1 promoter activity pilot screening. **b** ASS1 promoter activity in HEK293 cells treated with 10 μM decitabine for 12 hr or 24 hr by luciferase assay. **c** ASS1 and CHOP expression levels in Huh7 cells, SNU475, and SNU449 cells treated with decitabine indicated concentration. **d** Representative of cell viability in Huh7 cells with combination treatment of cisplatin and decitabine. **e, f** Cell survival rates of Huh7 and SNU449 cells after combination treatment with 0, 10, or 20 μM cisplatin and 10 μM decitabine by nuclei counting. **g** Expression of apoptosis related proteins; cleaved caspase 3 and PARP activation form expression levels in Huh7 and SNU475 cells after combination treatment with 0, 10, or 20 μM cisplatin and 10 μM decitabine. At least triplicate analysis in each set of experiment. ^*^P < 0.05 compared control group

We screened 3527 compounds selected from compound libraries (including LOPAC, Selleck anticancer and kinase inhibitors, FDA collection, and IND drugs) for drug repositioning in duplicate to confirm the reproducibility of observed effects. Compounds were screened at an initial concentration of 1 uM with a readout looking at an increase in ASS1 promoter activity. Positive and negative controls would be 10 nM TG and 0.01% dimethyl sulfoxide(DMSO). A Pearson correlation coefficient of 0.71 for replicate screens indicated that the assay was reliable (Fig. 7a). Fifteen compounds, including TG, constituted the primary hits that significantly elevated *ASS1* promoter activity (Additional file 4: Table S4). Through follow-up dose-response studies performed to quantify the potency of selected hits, we found that decitabine, a hypomethylating agent, most efficiently elevated *ASS1* promoter activity among the hits (Fig. 7b), although treatment with decitabine did not result in anti-HCC efficacy (Additional file 8: Fig. S4).

Furthermore, expression of both ASS1 and CHOP was significantly enhanced in the presence of decitabine in Huh7, SNU449, and SNU475 cells (Fig. 7c).

Next, we investigated whether increasing ASS1 expression via decitabine treatment improved efficacy of conventional chemotherapy against HCC. Treatment with decitabine significantly enhanced sensitivity to cisplatin in Huh7 (Fig. 7d, e) and SNU449 cells (Fig. 7f). Expression of apoptosis markers, such as cleaved PARP and caspase-3, was increased in HCC cells after treatment with cisplatin and decitabine in Huh7 and SNU 475 cells (Fig. 7g).

**Fig. 8:**
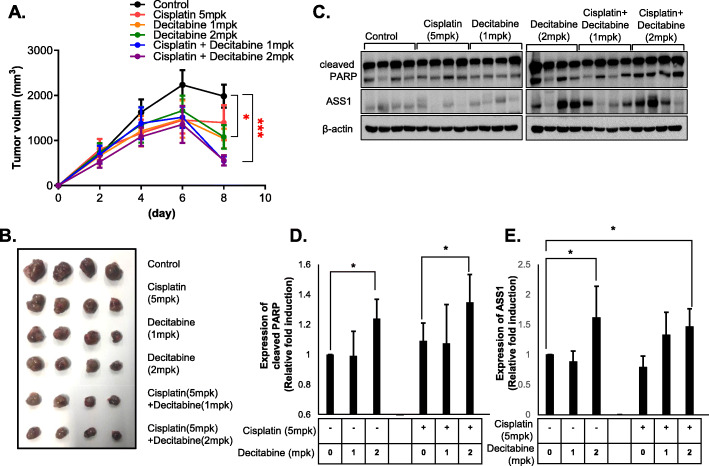
The combination treatment of cisplatin and decitabine improves efficacy of anti-HCC therapeutic in xenograft mice models **a** Tumor volume curves and **b** Representative of tumors from xenograft mice treated with cisplatin (5 mpk) and decitabine (1 or 2mpk) (*N* = 4). **c** PARP activation form (cleaved PARP) and ASS1 expression levels in tumor tissues of xenograft mice treated with cisplatin and decitabine by western blot assay. **d-e** Relative quantitative expression analysis of cleaved PARP and ASS1. ^*^P < 0.05 compared to control group

To determine whether decitabine could enhance the efficacy of anti-HCC therapies *in vivo*, we transplanted Huh7 cells into BALB/c mice. Administration of decitabine or cisplatin alone led to a subtle reduction of tumor growth or tumor regression, respectively. However, combination treatment with both agents significantly reduced tumor volume *vs.* treatment with cisplatin alone (Fig. 8a, b), suggesting that decitabine can act as a therapeutic adjuvant with cisplatin to treat HCC. Western analysis of tumor tissue showed that treatment with cisplatin plus decitabine increased levels of cleaved PARP relative to cisplatin alone (Fig. 8c, d) and administration of decitabine induced upregulation of ASS1 in HCC-implanted xenograft mice, as *in vitro* (Fig. 8c, e). These data suggest that decitabine might serve as a sensitizer for highly efficient treatment of HCC with cisplatin.

Taken together, our results show that treatment with decitabine increases ASS1 expression, thereby facilitating the robust therapeutic activity of combined decitabine and anti-HCC therapies like cisplatin.

## Discussion

HCC is the most common primary malignancy of the liver and progresses rapidly, leading to poor overall survival and, consequently, short treatment duration. Despite high-profile failures of multiple VEGFR-targeting agents against HCC, substantial research and development efforts continue in this area [21–24]. For example, sorafenib, a multi-kinase inhibitor, was approved for treating advanced HCC in 2007, [25], but its effectiveness and safety have not been extensively demonstrated [26]. Moreover, HCC rapidly becomes sorafenib-resistant [27]. Nevertheless, lenvatinib [28], regorafenib [29], ramucirumab [30] and cabozantinib [31], which recapitulate the mechanisms of sorafenib as VEGFR inhibitors, are expected to be approved for second-line treatment of advanced HCC in the United States and EU5, but not in the Asia-Pacific region. Unfortunately, the majority of HCC cases occur in the Asia-Pacific region, which highlights the serious challenge of HCC drug discovery, as clinical trials do not accurately reflect the epidemiology of this disease.

Currently, immunotherapy is a promising and alternative treatment that has been successful substituted for many different cancers including HCC [32]. Since HCC is an inflammation induced cancer, it is certainly an interesting target for an immune-based approach. In fact, many researchers suggested that the immunotherapy combination of immune checkpoint inhibitors for advanced HCC therapy [33]. The clinical trials results with immune checkpoint inhibitors have been interesting, which has already been approved by the FDA in US for the first-line treatment for patients with metastatic liver cancer [34].

To supplement the development of VEGF/VEGFR inhibitors for HCC therapy, new targets are currently being investigated, such as PD-1/PD-L1 [35, 36], CTLA-4 [37, 38], c-Met [39], TGF [40], PI3K/PTEN/Akt/mTOR [41], and Hedgehog [42] *etc*. Additionally, dysregulation of many signaling pathways has been linked to HCC development and progression, so researchers have sought to derive novel target genes and drug candidates related to these pathways. Recently, metabolic reprogramming of the tumor microenvironment through alterations in intracellular and extracellular metabolites and metabolic pathways have also been considered valuable targets for HCC therapy. Most tumor cells must adapt their metabolism to survive and proliferate in this dynamic and often harsh TME [43, 44]; thus, understanding the crosstalk between tumor cells and their m is needed to fully understand tumor development, progression, and chemoresistance in HCC. To avoid overlooking valuable drug targets due to incomplete recapitulation of the TME in experimental studies, we utilized a 3D culture system of HCC cells to identify such targets. Through proteomic analysis of HCC cells in 2D and 3D culture, we found that various types of stress in 3D HCC cultures impact energy metabolism, including expression of ASS1, which regulates arginine and citrulline metabolism (Fig. 1). Similar to our results, ASS1 was also elevated in mesothelioma 3D spheroids and in human pleural mesotheliomas, although mesothelioma is considered by many to be an ASS1-deficient tumor [45].

Because Korea ranks highest in the world in terms of liver cancer incidence and mortality rates, we also investigated the clinical value of ASS1 in Korean patients with HCC. Although most HCC samples exhibited low ASS1 expression, Korean HCC patients with increased ASS1 expression in their tumor tissue have a more favorable prognosis than those with lower expression levels (Fig. 2, Additional file 6: Fig. S2). ASS1 is differentially expressed across a wide range of tumors [46], the effects of which also differ in each type of tumor.

Because HCC tumors are auxotrophic for the essential amino acid arginine [47, 48], the depletion of which leads to tumor death, arginine depletion is a strategy for HCC therapy. HCCs exhibit loss of ASS1 expression via epigenetic silencing of CpG island methylation within the *ASS1* promoter [49], leading to auxotrophy [46, 50].

In this study, we sought to elucidate the novel functions of ASS1, which was upregulated in HCC spheroids. The role of ASS1 in tumor biology is unclear, as the protein may act either as a tumor suppressor [51–53] or as pro-metastatic or carcinogenic factor [13, 54, 55] in various carcinomas. Here, we demonstrated the role of ASS1 as a tumor suppressor in liver cancer. Specifically, ASS1 overexpression inhibited HCC spheroid growth and migration *in vitro.* As ASS1 positivity is a biomarker of cisplatin sensitivity, ASS1 overexpression also hypersensitized HCC cells to chemotherapeutic agents (Fig. 3, Additional file 2: Table S2, Additional file 3: Table S3).

Microarray analysis using HCC spheroids predicted the relationship between ER stress response and ASS1 expression in HCC spheroids. Arginine starvation induces ER stress in solid cancer cells [56], but roles of ASS1 in ER stress response have not been previously studied. As expected, ER stress in HCC spheroids not only increased ASS1 expression but also induced translocation of ASS1 from the cytoplasm to the ER (Fig. 4). Consequently, upregulated ASS1 in HCC cells facilitated ER stress-related cell death via induction of CHOP expression independently of arginine metabolism, as well as p53 activation and NO production (Fig. 6). Although ASS1 induced susceptibility to genotoxic stress as a p53-activated gene in colorectal cancer [52] and mediated fluid shear stress by NO production [57, 58], ER stress-related cell death in ASS1-overexpressing HCC cells is independent of p53 activation and NO production. Overexpression of ASS1 exhibited stronger tumor regression in HCC with mutant p53 than in HCC with wild-type p53. Identify critical pathway to inhibit mutant p53-specific survival and growth regulatory pathways are highly promising for effective treatment of many cancers, because mutant p53 often exhibits novel gain-of-functions to promote tumor growth and metastasis. Therefore, the increase of tumor suppression by ASS1-overexpression in p53 mutant HCC cells demonstrated that is a unique and valuable new pathway to overcome the p53-mutant specific survival pathways.

The ER stress-mediated upregulation of ASS1 in HCC spheroids restricted their growth through ER stress-induced apoptosis via increased expression of CHOP. Thus, we thought that targeting the upregulation of ASS1 may represent a promising strategy for HCC therapy. Indeed, treatment with the hypomethylating agent decitabine both enhanced ASS1 expression and improved the efficacy of cisplatin against HCC cells (Figs. 7 and 8).

## Conclusions

**Fig. 9:**
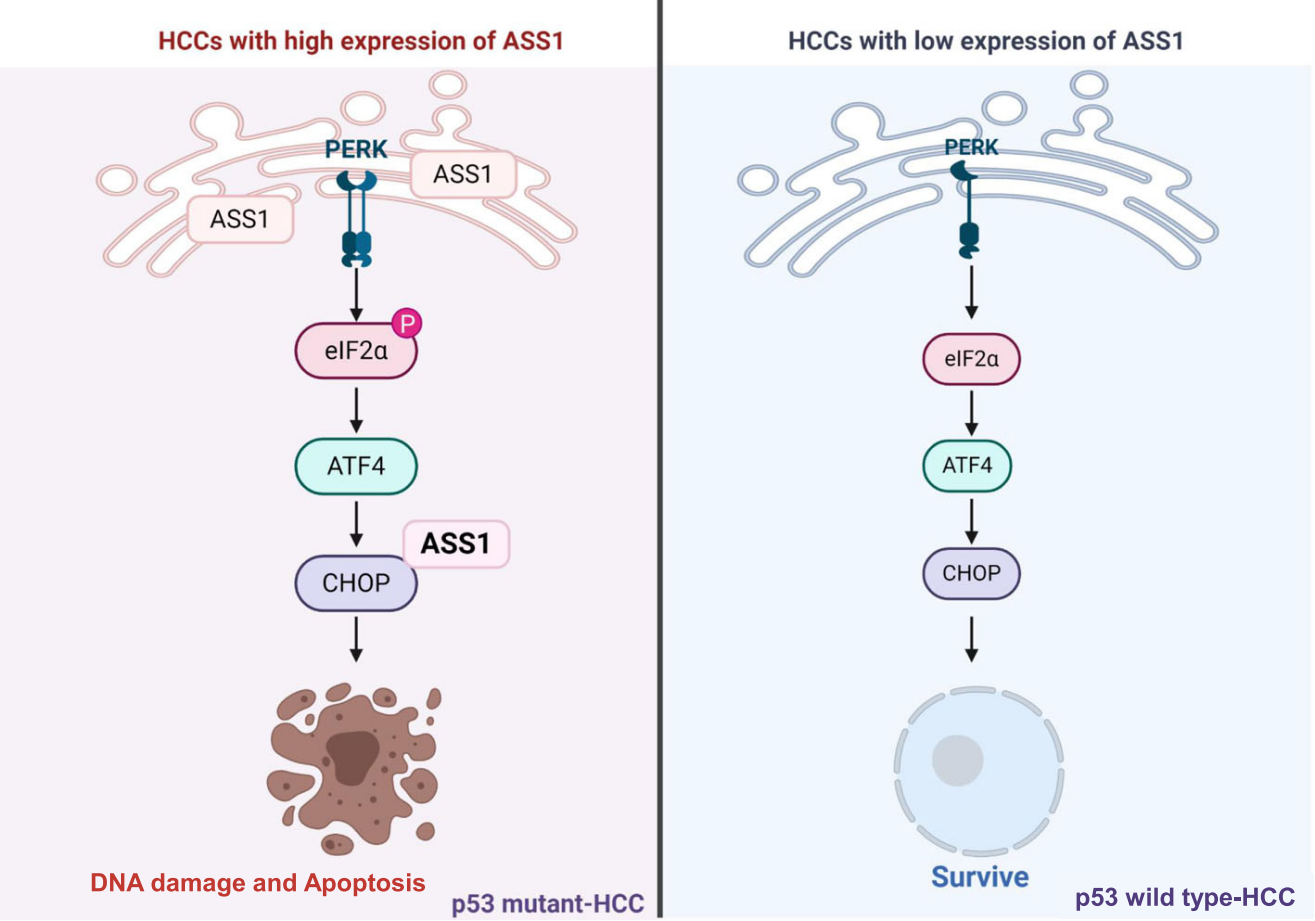
Graphical summary of novel functions of ASS1 as a tumor suppressor through ER stress-induced apoptosis in mutant p53 HCCs.

Our results provide clear evidence that the low ASS1 expression level in HCC tissue negatively impacted the overall survival of patients with liver cancer. Moreover, we discovered novel functions of ASS1 as a tumor suppressor through its arginine metabolism–independent facilitation of ER stress-induced apoptosis in mutant p53 HCCs (Fig. 9). Therefore, we conclude that identifying compounds that increase ASS1 expression may be a promising approach for enhancing HCC therapy.

## Supplementary Information

**Additional file 1: Table S1.** List of genes in ASS1 overexpressed HCC spheroids.

**Additional file 2: Table S2.** Changing of IC50 value by depletion of ASS1 (Unit: μM).

**Additional file 3: Table S3.** Changing of IC50 value by overexpression of ASS1 (Unit: μM).

**Additional file 4: Table S4.** List of primary hit compounds.

**Additional file 5: Figure S1.** Expression of identified proteins including Nucleophosmin, peroxiredoxin, Aldolase A and HSPD1 in monolayer (2D) and spheroids (3D).

**Additional file 6: Figure S2.** ASS1 expression in 58 Korean patients with nodular liver cancer and hepatitis B infection.

**Additional file 7: Figure S3.** EMT related proteins (α-SMA, N-cadherin, E-cadherin, Snail, and Vimentin), GRP78 and ASS1 in Huh cells after TG treatment.

**Additional file 8: Figure S4.** Cell viability analysis of decitabine treatment in Huh7 cells.

## Data Availability

Information is included in the Methods section.

## Abbreviations

ALDOA: Aldolase A
ASNS: Asparagine synthetase
ASS1: Argininosuccinate synthase 1
ATF3: Activating transcription factor 3
ATF4: Activating transcription factor 4
ATF6: Activating transcription factor 6
CALR: Calreticulin
CHOP: C/EBP Homologous Protein
cPTIO: Carboxy-PTIO
CTLA-4: Cytotoxic T-lymphocyte-associated protein 4
eIF2α: Eukaryotic initiation factor 2 α
ECM: Extracellular matrix
EMT: Epithelial-to-mesenchymal transition
ER: Endoplasmic reticulum
FDA: Food and drug administration
GRP78: Glucose-Regulated Protein 78
HCC: Hepatocellular carcinoma
HSF5: Heat shock transcription factor 5
HSP90B1: Heat shock protein 90 beta family member 1
HSPA1A: Heat shock protein family A member 1A
HSPA5: Heat shock protein family A member 5
HSPD1: Heat shock protein family D member 1
IF: Immunofluorescence
IND: Investigational new drug application; α-SMA
IRE1: Inositol-requiring enzyme-1
LOPAC: Library of Pharmacologically Active Compounds
mTOR: Mammalian target of rapamycin
NO: Nitric oxide
PARP: Poly ADP-ribose polymerase
PD-1: Programmed cell death protein 1
PD-L1: Programmed death-ligand 1
PERK: PKR-like ER kinase
PI3K: Phosphoinositide 3-kinases
PTEN: Phosphatase and tensin homolog
S1P: Site-1 protease
S2P: Site-2 protease
TCGA: The Cancer Genome Atlas
TG: Thapsigargin
TGF: Transforming growth factor
TM: Tunicamycin
TME: Tumor microenvironment
UPR: Unfolded protein response
VEGF: Vascular endothelial growth factor
VEGFR: Vascular endothelial growth factor receptor
XBP1: X-box binding protein 1
