# Supplementary material for: Argininosuccinate synthase 1 suppresses tumor progression through activation of PERK/eIF2α/ATF4/CHOP axis in hepatocellular carcinoma
Source: J Exp Clin Cancer Res. 2021 Apr 10;40:127. doi: 10.1186/s13046-021-01912-y (PMC8035787; doi:10.1186/s13046-021-01912-y)
Supplement: Supplementary file 1 — Additional file 1: Table S1. List of genes in ASS1 overexpressed HCC spheroids. [file 13046_2021_1912_MOESM1_ESM.docx]

**Additional file: Table S1. List of genes in ASS1 overexpressed HCC spheroids.**

| **List of Gene** |
| --- |
| ASNS |
| ATF3 |
| CD68 |
| CGGBP1 |
| DDIT3 |
| DNAJC12 |
| GAD1 |
| GDF15 |
| HAX1 |
| HSPA1A |
| PCK2 |
| SAT1 |
| YRDC |
