# Supplementary material for: Argininosuccinate synthase 1 suppresses tumor progression through activation of PERK/eIF2α/ATF4/CHOP axis in hepatocellular carcinoma
Source: J Exp Clin Cancer Res. 2021 Apr 10;40:127. doi: 10.1186/s13046-021-01912-y (PMC8035787; doi:10.1186/s13046-021-01912-y)
Supplement: Supplementary file 2 — Additional file 2: Table S2. Changing of IC50 value by depletion of ASS1 (Unit: μM). [file 13046_2021_1912_MOESM2_ESM.docx]

**Additional file: Table S2.** **Changing of IC_50_ value by depletion of ASS1** (Unit: μM)

|  | **Hep3B SiCont** | **Hep3B SiASS1** | **PLC SiCont** | **PLC SiASS1** |  |  |
| --- | --- | --- | --- | --- | --- | --- |
| Cisplatin | 4.231 | 9.372 | 1.477 | 2.64 |  |  |
| 5-FU | 0.5397 | 2.799 | 1.242 | 3.186 |  |  |
| Sorafenib | 12.9 | 19.74 | 11.27 | 60.5 |  |  |
