# Supplementary material for: Argininosuccinate synthase 1 suppresses tumor progression through activation of PERK/eIF2α/ATF4/CHOP axis in hepatocellular carcinoma
Source: J Exp Clin Cancer Res. 2021 Apr 10;40:127. doi: 10.1186/s13046-021-01912-y (PMC8035787; doi:10.1186/s13046-021-01912-y)
Supplement: Supplementary file 3 — Additional file 3: Table S3. Changing of IC50 value by overexpression of ASS1 (Unit: μM). [file 13046_2021_1912_MOESM3_ESM.docx]

**Additional file: Table S3.** **Changing of** **IC_50_ value by overexpression of ASS1** (Unit: μM)

|  | **Huh7 Cont** | **Huh7 ASS1** | **SNU475 Cont** | **SNU475 ASS1** |
| --- | --- | --- | --- | --- |
| Cisplatin | 11.63 | 6.521 | 6.42 | 0.8177 |
| 5-FU | 13.11 | 7.99 | 10.03 | 3.065 |
| Sorafenib | 6.163 | 2.253 | 4.209 | 2.083 |
