# Supplementary material for: Argininosuccinate synthase 1 suppresses tumor progression through activation of PERK/eIF2α/ATF4/CHOP axis in hepatocellular carcinoma
Source: J Exp Clin Cancer Res. 2021 Apr 10;40:127. doi: 10.1186/s13046-021-01912-y (PMC8035787; doi:10.1186/s13046-021-01912-y)
Supplement: Supplementary file 4 — Additional file 4: Table S4. List of primary hit compounds. [file 13046_2021_1912_MOESM4_ESM.docx]

**Additional file: Table S4. List of primary hit compounds**

|  |  |  |
| --- | --- | --- |
|  | **Name of compounds** | **Mechanism of action** |
| 1 | PFI-1 | Selective BET inhibitor |
| 2 | (+/-)-JQ1 | BET inhibitor |
| 3 | HPI-1 hydrate | A Hedgehog pathway inhibitor; Gli1/Gli2 inhibitor. |
| 4 | Decitabine | hypomethylating agent |
| 5 | Pimecrolimus | Immunomodulating agent of the calcineurin inhibitor |
| 6 | JNJ-7706621 | Dual inhibitor of CDKs and aurora kinases |
| 7 | Thapsigargin | Inhibitor of theendoplasmic reticulum Ca2^+^ ATPase |
| 8 | PIK-93 | PI4K (PI4KIIIβ) inhibitor |
| 9 | TG101348 (SAR302503) | Inhibitor of JAK2 |
| 10 | Adaphostin | p210 bcr/abl tyrosine kinase inhibitor |
| 11 | BMS-345541 | Primary target of IKK-2 |
| 12 | Clobetasol propionate | Corticosteroid |
| 13 | NU7441(KU-57788) | Selective inhibitor of DNA-PK |
| 14 | Erlotinib HCl | EGFR inhibitor |
| 15 | Deforolimus (Ridaforolimus) | mTOR inhibitor |
