# Supplementary figures and images for: Argininosuccinate synthase 1 suppresses tumor progression through activation of PERK/eIF2α/ATF4/CHOP axis in hepatocellular carcinoma
Source: J Exp Clin Cancer Res. 2021 Apr 10;40:127. doi: 10.1186/s13046-021-01912-y (PMC8035787; doi:10.1186/s13046-021-01912-y)

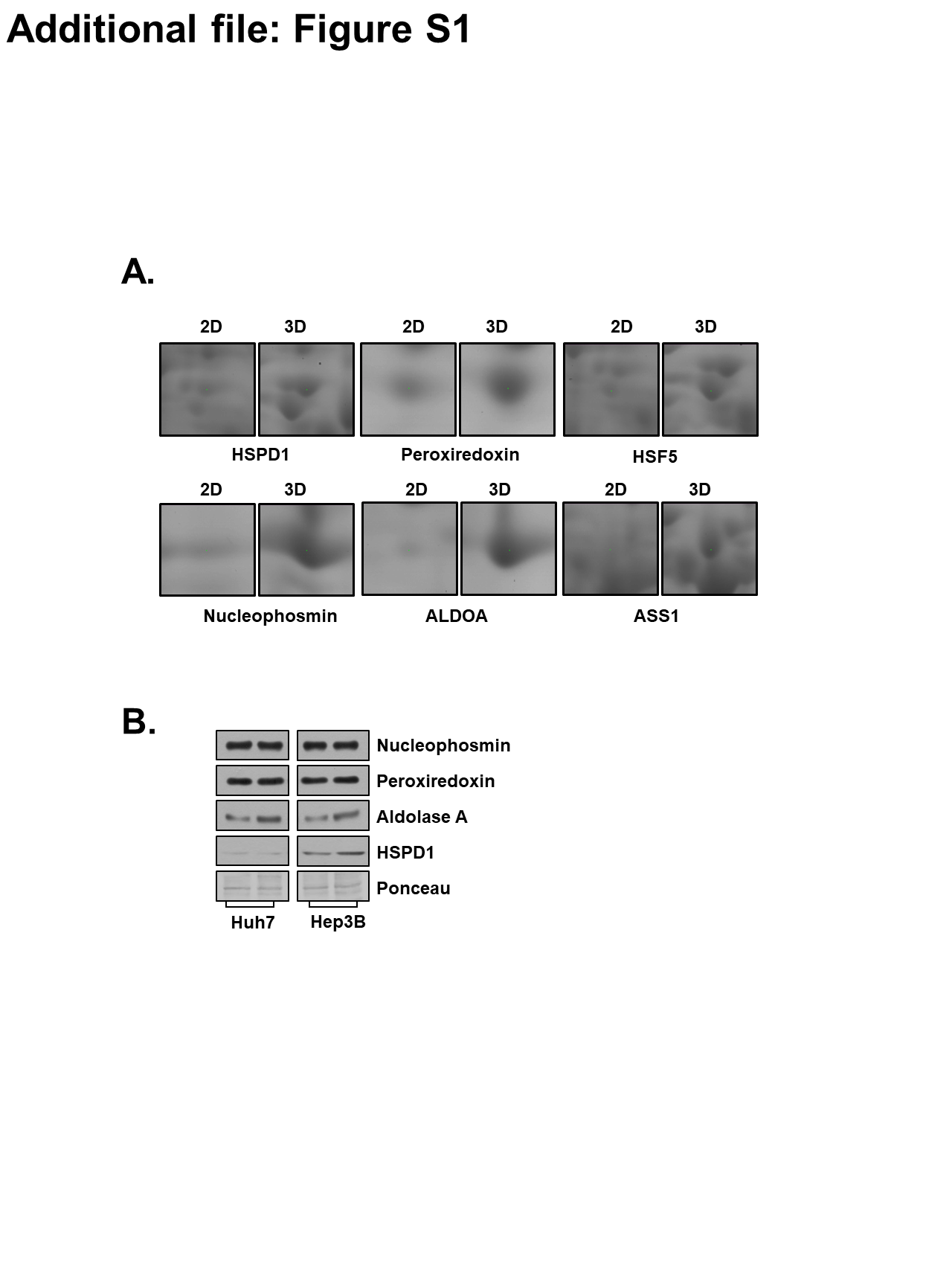

Supplement: Supplementary file 5 — Additional file 5: Figure S1. Expression of identified proteins including Nucleophosmin, peroxiredoxin, Aldolase A and HSPD1 in monolayer (2D) and spheroids (3D). [file 13046_2021_1912_MOESM5_ESM.tif]

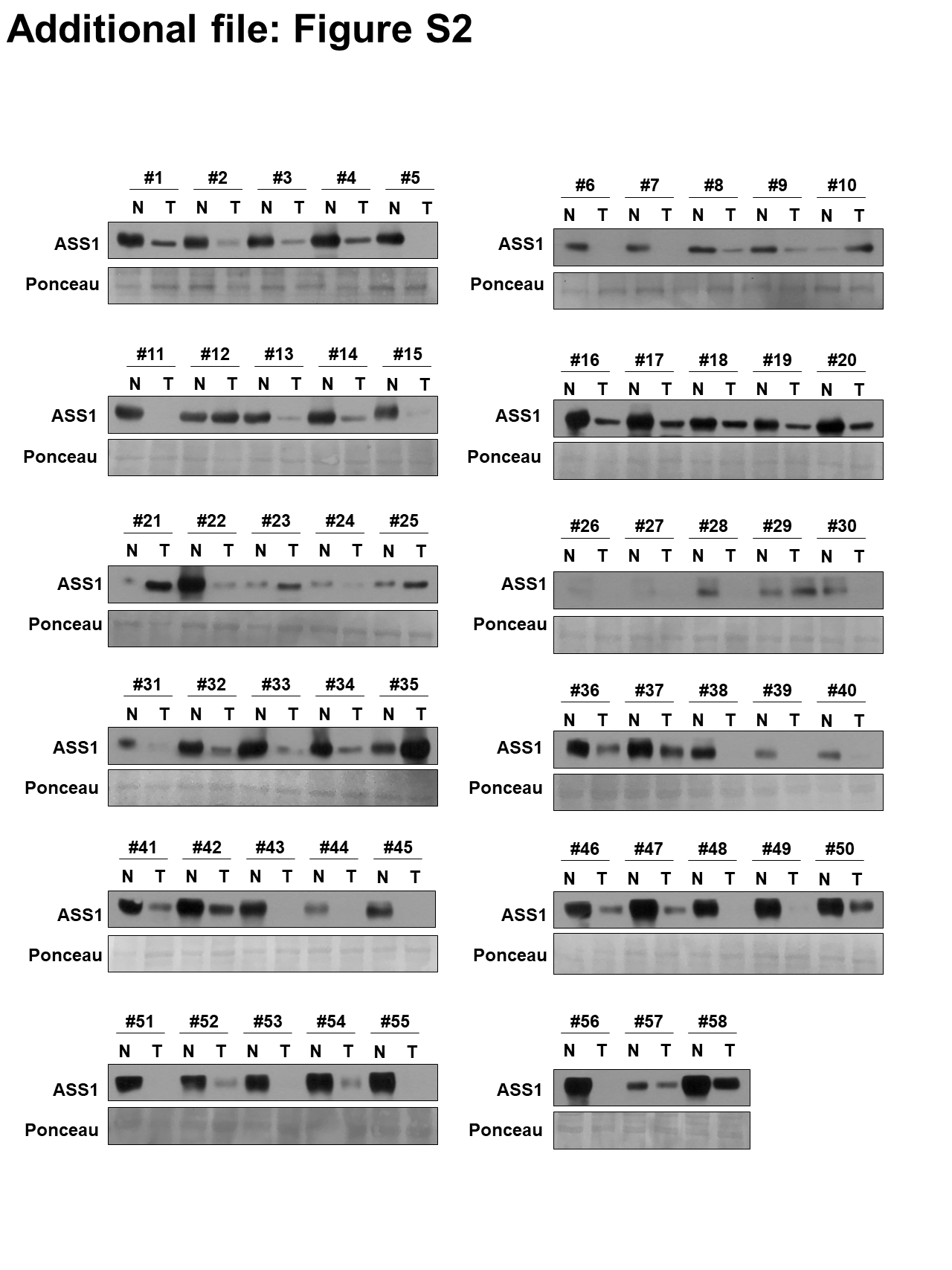

Supplement: Supplementary file 6 — Additional file 6: Figure S2. ASS1 expression in 58 Korean patients with nodular liver cancer and hepatitis B infection. [file 13046_2021_1912_MOESM6_ESM.tif]

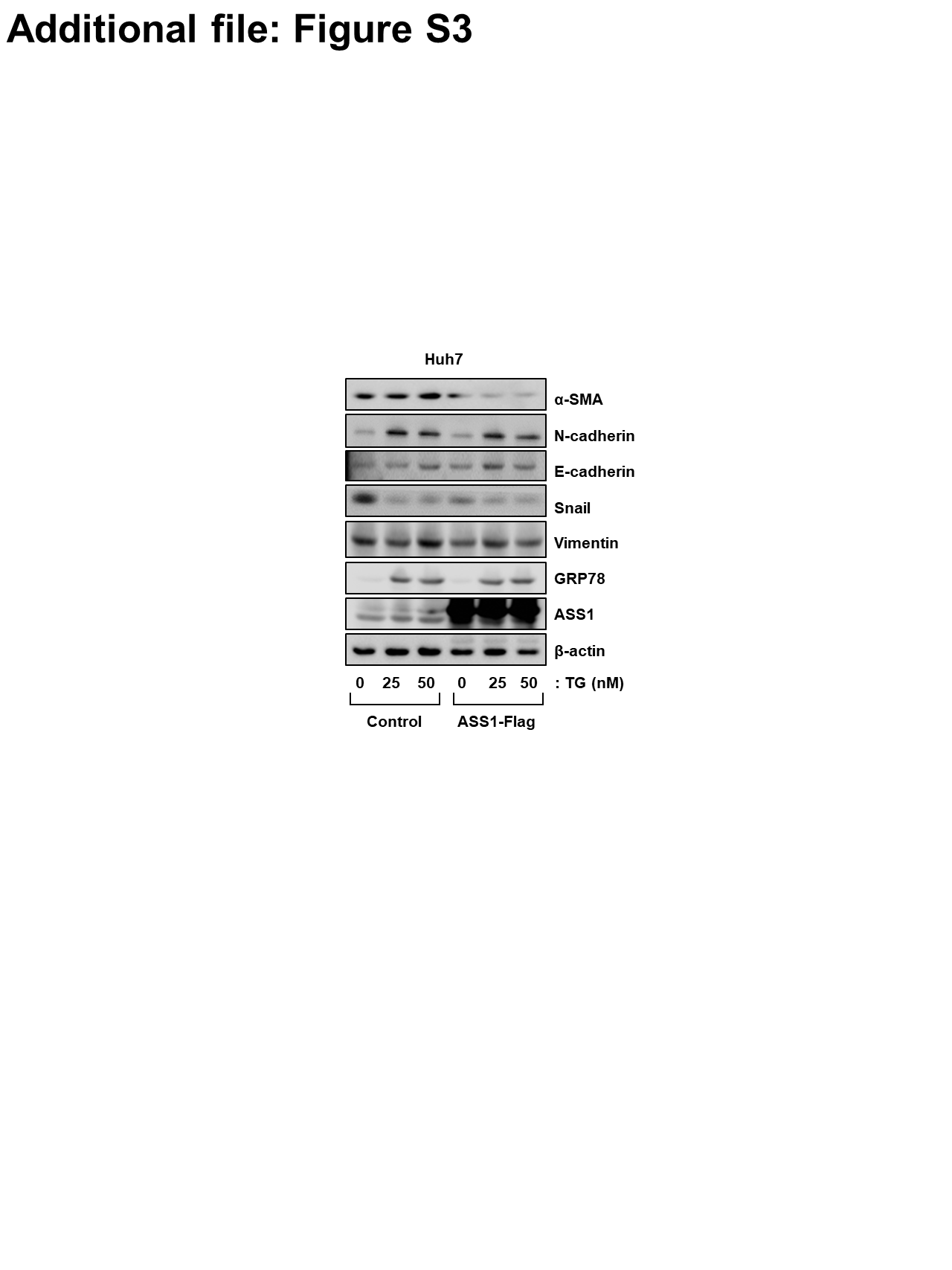

Supplement: Supplementary file 7 — Additional file 7: Figure S3. EMT related proteins (α-SMA, N-cadherin, E-cadherin, Snail, and Vimentin), GRP78 and ASS1 in Huh cells after TG treatment. [file 13046_2021_1912_MOESM7_ESM.tif]

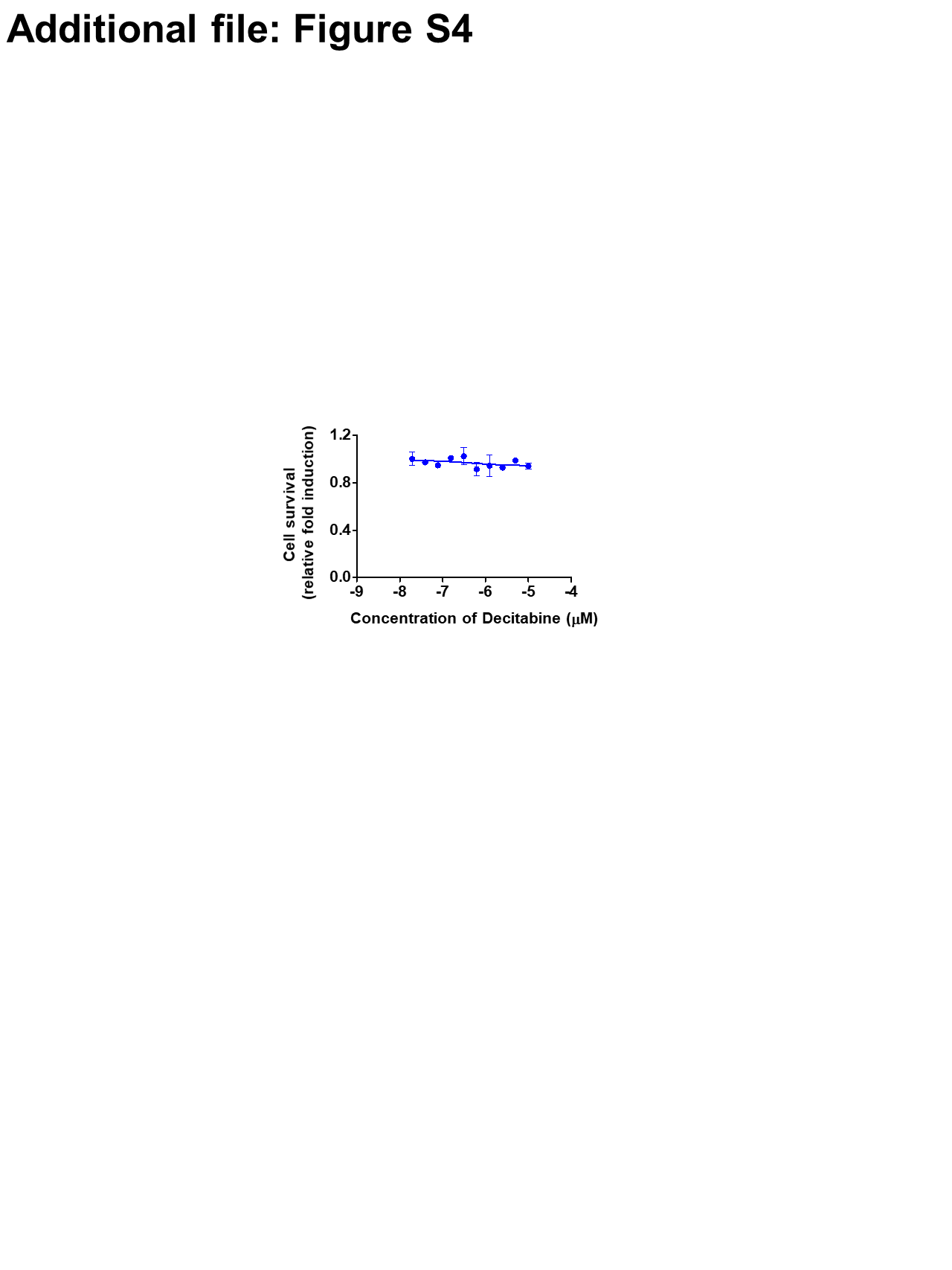

Supplement: Supplementary file 8 — Additional file 8: Figure S4. Cell viability analysis of decitabine treatment in Huh7 cells. [file 13046_2021_1912_MOESM8_ESM.tif]
